# RND-type drug eﬄux pumps from Gram-negative bacteria: molecular mechanism and inhibition

**DOI:** 10.3389/fmicb.2015.00377

**Published:** 2015-04-28

**Authors:** Henrietta Venter, Rumana Mowla, Thelma Ohene-Agyei, Shutao Ma

**Affiliations:** ^1^School of Pharmacy and Medical Sciences, Sansom Institute for Health Research, University of South AustraliaAdelaide, SA, Australia; ^2^Department of Pharmacology, University of CambridgeCambridge, UK; ^3^Department of Medicinal Chemistry, School of Pharmaceutical Sciences, Shandong UniversityJinan, China

**Keywords:** multidrug resistance, drug eﬄux, eﬄux pump inhibitor, Gram-negative, pathogen, antimicrobial resistance

## Abstract

Drug eﬄux protein complexes confer multidrug resistance on bacteria by transporting a wide spectrum of structurally diverse antibiotics. Moreover, organisms can only acquire resistance in the presence of an active eﬄux pump. The substrate range of drug eﬄux pumps is not limited to antibiotics, but it also includes toxins, dyes, detergents, lipids, and molecules involved in quorum sensing; hence eﬄux pumps are also associated with virulence and biofilm formation. Inhibitors of eﬄux pumps are therefore attractive compounds to reverse multidrug resistance and to prevent the development of resistance in clinically relevant bacterial pathogens. Recent successes on the structure determination and functional analysis of the AcrB and MexB components of the AcrAB-TolC and MexAB-OprM drug eﬄux systems as well as the structure of the fully assembled, functional triparted AcrAB-TolC complex significantly contributed to our understanding of the mechanism of substrate transport and the options for inhibition of eﬄux. These data, combined with the well-developed methodologies for measuring eﬄux pump inhibition, could allow the rational design, and subsequent experimental verification of potential eﬄux pump inhibitors (EPIs). In this review we will explore how the available biochemical and structural information can be translated into the discovery and development of new compounds that could reverse drug resistance in Gram-negative pathogens. The current literature on EPIs will also be analyzed and the reasons why no compounds have yet progressed into clinical use will be explored.

## Introduction

Over the last two decades there has been a dramatic surge in the number of multidrug resistant bacteria, yet paradoxically the number of pharmaceutical companies developing new antimicrobial agents has dwindled during this same period. As a result, antibiotic resistance is now one of the world’s most pressing health problems (WHO, 2014). Therefore, new treatments to combat drug resistant bacteria are urgently needed if we do not want to return to the high mortality rates associated with infections during the pre-antibiotic era (Bush et al., 2011; WHO, 2014).

Hospital acquired pathogens such as *Staphylococcus aureus*, *Klebsiella pneumonia*, *Acinetobacter baumannii,* and *Pseudomonas aeruginosa* which can cause life-threatening infections display high levels of antibiotic resistance (Poole, 2011; Bassetti et al., 2013). Resistance of *K. pneumonia* to carbapenems, the last resort treatment for severe infections, of up to 54% of cases were reported (WHO, 2014).

Recently a few new antibiotics have been approved for the use against Gram-positive organisms (Butler and Cooper, 2011). However, infections caused by Gram-negative pathogens proved much harder to treat due to the very high intrinsic drug resistance displayed by Gram-negative organisms. This intrinsic drug resistance is due to presence of an outer membrane which acts as a permeability barrier and by the expression of drug eﬄux pumps.

Drug eﬄux pumps are protein complexes which reside in the membrane and remove antimicrobials and toxins, thereby lowering their concentration inside the cell to sub-toxic levels (Poole, 2004, 2005; Piddock, 2006a; Nikaido and Pages, 2012). These proteins recognize and expel a wide range of structurally diverse antibiotics with different mechanisms and sites of action. The clinical implication of this substrate promiscuity is the development of multidrug resistance where a pathogen displays resistance against multiple classes of antimicrobials.

Apart from antibiotics drug eﬄux proteins can also transport antiseptics and disinfectants (Chuanchuen et al., 2003; Sanchez et al., 2005; Mima et al., 2007; Pumbwe et al., 2007), detergents (including naturally occurring bile salts; Rosenberg et al., 2003; Lin et al., 2005), fatty acids (Lee and Shafer, 1999; Lennen et al., 2013), heavy metals (Silver and Phung, 1996; Walmsley and Rosen, 2009), solvents (White et al., 1997; Ramos et al., 2002; Segura et al., 2012), and virulence factors (Piddock, 2006b). Therefore, drug eﬄux pumps are also important constituents of bacterial pathogenesis, virulence, and biofilm formation (Hirakata et al., 2002, 2009; Piddock, 2006b; Ikonomidis et al., 2008; Martinez et al., 2009; Baugh et al., 2012, 2014; Amaral et al., 2014). In addition, micro-organisms can only acquire resistance in the presence of drug eﬄux pumps (Lomovskaya and Bostian, 2006; Ricci et al., 2006; Zhang et al., 2011; Piddock, 2014) as these non-specific pumps remove most compounds until the organism has had time to acquire resistance to an antibiotic through more specific adaptive mechanisms.

Despite their crucial role in bacterial pathogenesis and multidrug resistance there are currently no inhibitors for drug eﬄux pumps in clinical use. Therefore drug eﬄux pumps are attractive targets for inhibition. Eﬄux pump inhibitors (EPIs) will (a) synergise with currently used antibiotics, (b) restore the efficacy of antibiotics to which resistance has arisen, (c) reduce the incidence of emergence of drug-resistant pathogens, (d) reduce the ability of pathogens to infect the host as the inhibition of eﬄux attenuates the bacterium, and (e) prevent the development of highly drug resistant biofilms

## Drug Eﬄux Pumps in Gram-Negative Bacteria

**FIGURE 1:**
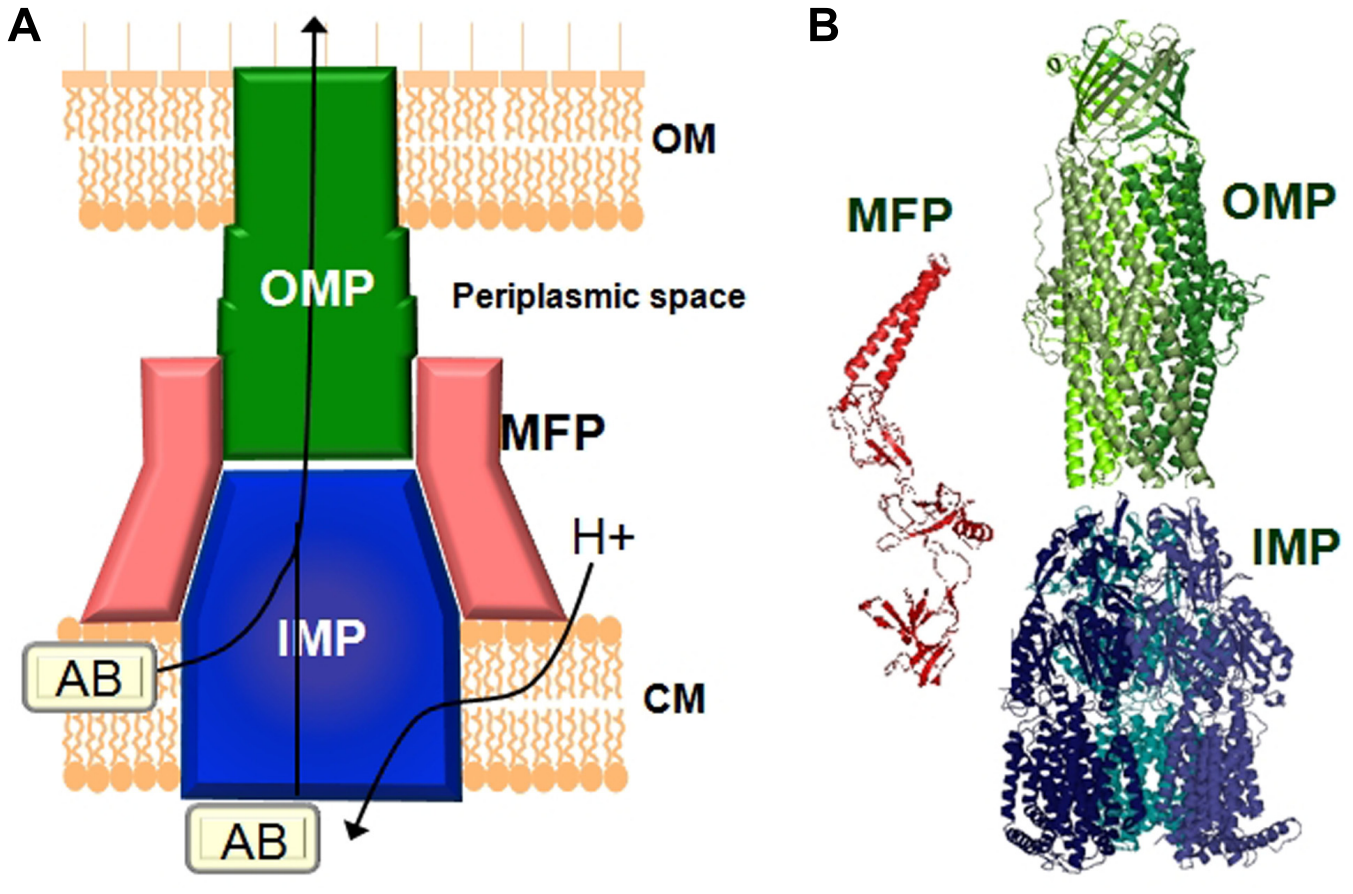
**Schematic representation 48.5pcof a tripartite drug eﬄux complex. (A)** The complex 48.5pcconsists of three proteins which span the inner-membrane (CM), the outer membrane (OM), and the periplasmic space. The inner-membrane protein (IMP), e.g., AcrB or MexB is responsible for substrate specificity and catalyzes ΔpH dependent drug transport. Examples of the outer membrane protein (OMP) are TolC or OprM. The periplasmic membrane fusion protein (MFP), e.g., AcrA or MexA connects the IMP and the OMP. **(B)** Structures of the individual components of the eﬄux pump. The MexA (pdb: 2V4D), MexB (pdb: 2V50), and OprM (pdb: 1WP1) proteins from *Pseudomonas aeruginosa* are given as examples.

Gram-negative pathogens rely on tripartite protein assemblies that span their double membrane to pump antibiotics from the cell. The tripartite complex consists of an inner membrane protein (IMP) of the resistance nodulation cell division (RND) family, an outer-membrane protein (OMP), and a periplasmic membrane fusion protein (MFP) which connect the other two proteins (**Figure 1**). The inner-membrane protein catalyses drug/H^+^ antiport and is the part of the complex responsible for drug selectivity. The best studied tripartite drug eﬄux complexes are the AcrA-AcrB-TolC and MexA-MexB-OprM transporters from *Escherichia coli* and *P. aeruginosa,* respectively, (Du et al., 2013). The IMPs AcrB and MexB share 86% similarity and MexB can functionally substitute for AcrB (Krishnamoorthy et al., 2008; Welch et al., 2010). The asymmetric structure of the AcrB homotrimer and subsequent biochemical analysis revealed a functional rotating mechanism where the monomers cycle through the different states loose (L), tight (T), and open (O; Murakami et al., 2006; Seeger et al., 2006, 2008b). IMPs such as AcrB consist of a transmembrane domain and periplasmic domain. The drug eﬄux pathway from the periplasm/outer membrane leaflet through the periplasmic domain of AcrB has been the focus of many studies and are now relatively well-understood (Murakami, 2008; Seeger et al., 2008a; Eicher et al., 2009; Misra and Bavro, 2009; Nikaido and Takatsuka, 2009; Pos, 2009; Nikaido, 2011; Nikaido and Pages, 2012; Ruggerone et al., 2013a,b). Recently, it was also found that mutations at the cytoplasmic face of MexB affected transport of drugs with targets inside the cell (Ohene-Agyei et al., 2012). This raises the possibility that similar to the cytoplasmic pathway for Cu(II) in CusA (Delmar et al., 2014), MexB might also have the ability to remove antibiotics from the inner membrane leaflet/cytoplasm (Ohene-Agyei et al., 2012). Targeted geometric simulations showed that such a cytoplasmic pathway could be possible even though it would not necessarily out-compete the periplasmic channel for drug binding and transport (Phillips and Gnanakaran, 2015). Biochemical and structural analysis revealed that the perplasmic binding site in AcrB contains a shallow (proximal) and deep (distal) binding pocket separated by a switch loop (G-loop) consisting of residues 614–621 (Nakashima et al., 2011; Eicher et al., 2012; Cha et al., 2014). Conformational flexibility in this loop is necessary to move the substrate along the extended binding site. Mutations that change the small glycine residues in this loop to bulkier residues affects transport of larger macrolide antibiotics such as erythromycin while the activity toward smaller compounds such as novobiocin, ethidium, and chloramphenicol remained unaffected (Bohnert et al., 2008; Wehmeier et al., 2009; Nakashima et al., 2011, 2013; Eicher et al., 2012). Therefore, EPIs would most effectively inhibit the eﬄux of different antibiotics by interaction with the switch loop.

Due to the complexity of these macromolecular structures progress on elucidating their assembly and structure was slow. Only very recently Du et al. (2014) used a creative approach of genetic fusion proteins to solve the first structure of a partially active, fully assembled, tripartite pump in the presence of a modulatory partner. This structure of AcrA–AcrB–AcrZ–TolC shed light on long disputed subunit stoichiometries and revealed that the complex assembles in a 3 : 6 : 3 ratio of AcrB : AcrA : TolC with one monomer of AcrZ bound to each subunit of AcrB. The role of the small protein AcrZ is not clear, however, as it alters the substrate specificity of AcrB (Hobbs et al., 2012) it most likely plays a modulatory role.

The structural similarity between transporters from different Gram-negative organisms means that EPIs developed against, e.g., the AcrA–AcrB–TolC eﬄux pump from *E. coli* would most likely be effective against other pathogens also. Our current understanding of the structure and function of RND eﬄux pumps from Gram-negative bacteria could therefore provide the basis for the informed and efficient design of inhibitors against these protein complexes.

## Approaches to Inhibit Drug Eﬄux

**FIGURE 2:**
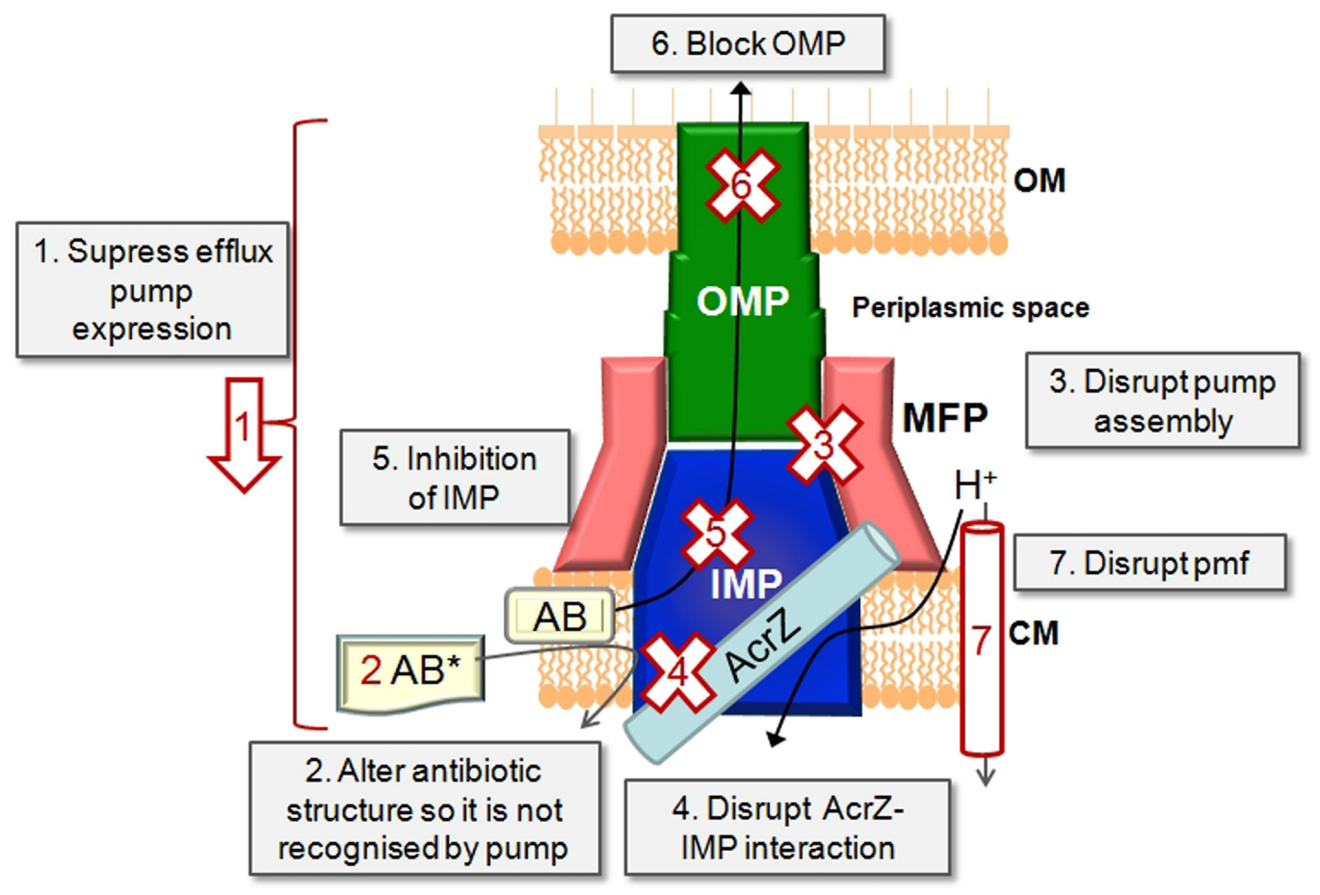
**Inhibition strategies**. Schematic representation of a tripartite drug eﬄux complex in complex with a small protein such as AcrZ. The possible approaches of inhibiting drug eﬄux are depicted.

The expression, function and assembly of drug eﬄux pumps of the RND class can be targeted in several ways (**Figure 2**).

### Targeting the Regulatory Network that Controls the Expression of Eﬄux Pumps as Levels of Pump Expression are Controlled by Activators and Repressors

Some progress has already been made in understanding the regulation of eﬄux pump expression, e.g., expression of AcrB from *Salmonella enterica* (Blair et al., 2014) and the regulation of eﬄux pump expression in *P. aeruginosa* (Wilke et al., 2008; Starr et al., 2012; Hay et al., 2013; Purssell and Poole, 2013; Lau et al., 2014). The expression levels of eﬄux pumps could be measured by real time PCR or with green fluorescent protein reporter fusions (Bumann and Valdivia, 2007; Ricci et al., 2012). Both these methods are amenable to high-throughput processing.

### Changing the Molecular Design of Old Antibiotics so that they are No Longer Recognized and Transported by the Eﬄux Pump

Given the wide range of compounds which could be recognized by drug eﬄux transporters, the plasticity in the binding sites, and the redundancy in aromatic residues in the binding pocket which could stabilize substrate binding (Du et al., 2013), this approach might prove a daunting task. In addition, altering the chemical structure of the antibiotic might render it less efficient against its intended cellular target. However, some progress has been made in this regard for a different class of drug eﬄux protein, the ATP binding cassette transporter, human P-glycoprotein where the substrate taxol was chemically modified so that P-glycoprotein no longer recognized it. This allowed the drug to cross the blood brain barrier and access its target receptor without being removed by P-glycoprotein (Rice et al., 2005).

### Preventing the Assembly of the Eﬄux Pump Components into a Functional Tripartite Pump by Targeting Protein–Protein Interfaces

This is a very promising approach which is still under-developed due to the lack of information of how tripartite pumps assemble. However, Tikhonova et al. (2011) showed that designed ankyrin repeat proteins (DARPins) could inhibit AcrAB-TolC function by inhibiting the interaction between AcrA and AcrB. The recent structure of a complete tripartite drug eﬄux pump and the information gained from that also opens up exciting new possibilities (Du et al., 2014). The interaction of purified protein components of the pump with each other can be measured with surface plasmon resonance (SPR). The ability of eﬄux pumps to assemble *in vivo* can be measured by cross-linking in whole cells with subsequent co-purifying of the pump components (Welch et al., 2010).

### Disrupting the Interaction Between AcrB and AcrZ

The exact role of AcrZ in drug eﬄux is still ill-defined. However, as AcrA–AcrB–TolC has a diminished ability to confer resistance to some drugs in the absence of AcrZ (Hobbs et al., 2012), this approach could be promising for restoring sensitivity to some antibiotics. Homologs of AcrZ are found in most Gram-negative bacteria, therefore the modulatory effect of RND class of transporters by small proteins is probably a widely conserved occurrence. The interaction between the IMP and a small protein such as AcrZ could be measured with SPR or with cross-linking in cells as mentioned above.

### Directly Blocking the IMP with a High Affinity Competing Substrate or Trapping the IMP in an Inactive Conformation

The recent crystal structure of AcrB and MexB bound to an inhibitor (Nakashima et al., 2013) and the advances in our understanding of how drugs are bound makes this option very attractive (see Eﬄux Pump Inhibitors Against Gram-Negative Bacteria Identified So Far). The ability of compounds to inhibit antibiotic eﬄux can be measured using drug accumulation or drug eﬄux assays (see Inhibition of Substrate Transport), while direct interaction between the test compound and the IMP component could be determined with isothermal calorimetry (ITC) or SPR (Tikhonova et al., 2011).

### Blocking the Exit Duct (the OMP)

A set of indole derivatives was designed based on the structure of TolC. These compounds were able to synergise with antibiotics and were reported to act on TolC specifically, presuming by preventing opening of the channel (Zeng et al., 2010). In addition, TolC from *E. coli* contains an electronegative entrance formed by an aspartate ring which is widely conserved throughout the TolC family and which could be a target for blocking by large cations (Andersen et al., 2002). The biggest challenge with this approach is achieving selectivity to the bacterial pores. Blocking of the OMP could be detected by inhibition of antibiotic eﬄux through the tripartite pump or by disruption of TolC-mediated conductance.

### Depleting the IMP From the Energy Needed to Drive the Drug/H^+^ Antiport Reaction

The proton motive force (pmf) can easily be disrupted by the use of ionophores or compounds that disrupt the membrane integrity in one way or another. However, these effects are mostly not specific for bacterial membranes and hence compounds that act in this way would be cytotoxic to the host cells too. The magnitude of the pmf and the effect of test compounds on these could be determined by the use of fluorescent probes specific for the ΔΨ or ΔpH components of the pmf (Venter et al., 2003).

## How Could EPIs be Identified?

**FIGURE 3:**
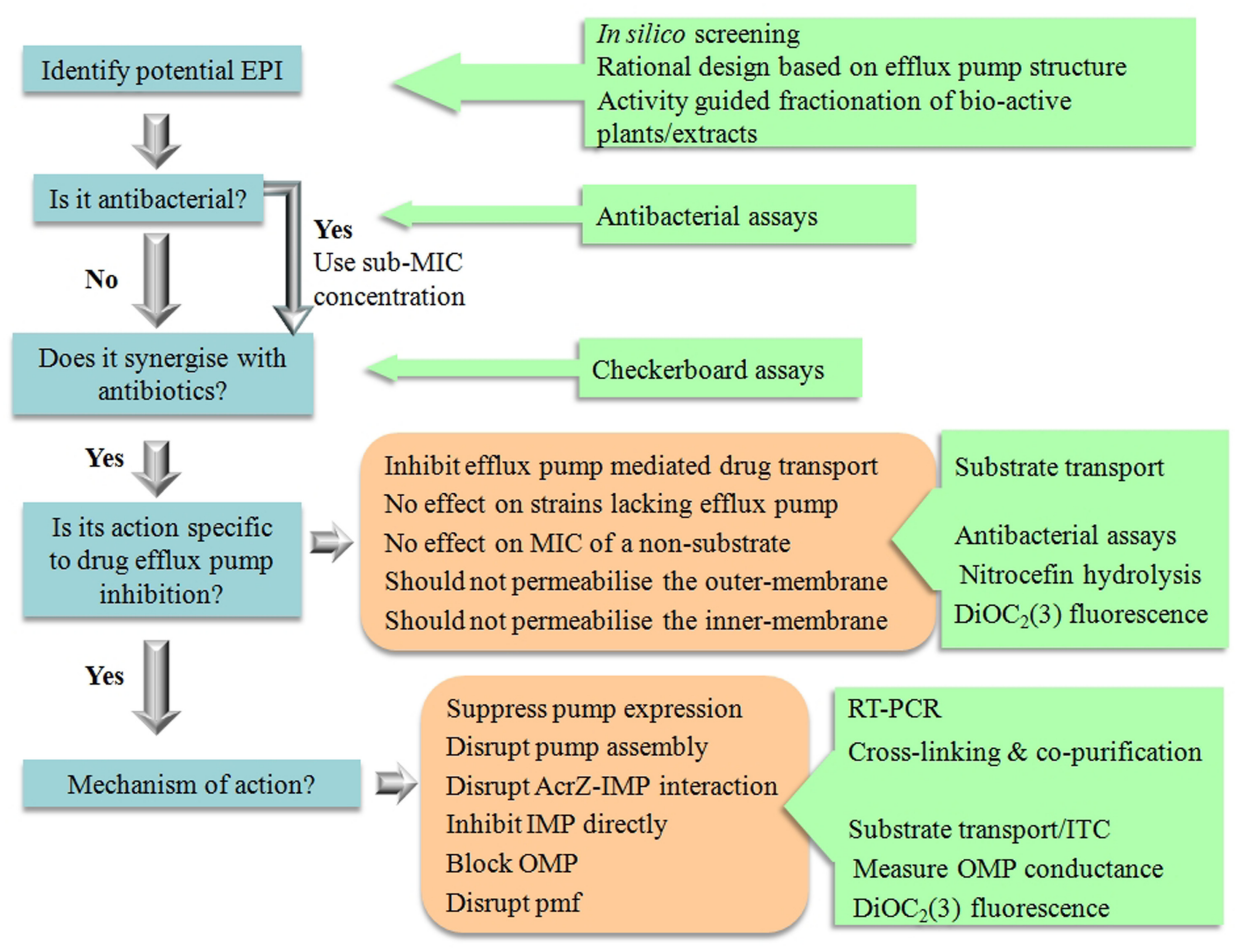
**The tools for EPI discovery**.

Significant effort went into the biochemical and structural characterization of drug eﬄux proteins from Gram-negative bacteria. Recent successes such as the structural determination of an intact pump and of IMPs bound to an inhibitor (Nakashima et al., 2013; Du et al., 2014) offer a solid platform for the rational design of EPIs using quantitative structure-activity relationship data (Ruggerone et al., 2013a; Wong et al., 2014; **Figure 3**).

Recently we used *in silico* screening to identify compounds which would bind to AcrB with reasonable affinity. Of the roughly fifty compounds docked, six compounds were selected for further study. The docking allowed us to provide an order of efficiency of the compounds as potential EPIs. The biochemical data compared well with the predictions from the docking showing that *in silico* screening could be used as an effective screening tool to limit the amount of experiments needed or save on precious and hard earned purified natural products (Ohene-Agyei et al., 2014).

Another approach with good scope for success is investigating compounds purified from plants (Tegos et al., 2002). Traditional peoples have used plants to treat infections for 100s if not 1000s of years. In western medicine, plants are thus far an under-utilized source of chemical components in the treatment of infectious disease. Resistance to medicinal plant extracts have not been described yet and extracts of herbal medicines have been shown to potentiate antibiotic action in resistant pathogens (Garvey et al., 2011; Ohene-Agyei et al., 2014). Therefore, it is likely that as well as antibacterial chemicals, plants may also produce compounds that circumvent eﬄux-mediated resistance. Hence, activity guided fractionation can be used to identify the bio-active phytochemicals in plant extracts with EPI activity against Gram-negative organisms (Garvey et al., 2011).

## Tools for Studying Eﬄux Pump Inhibitors

The most significant problem in current screening campaigns for EPIs is that in many cases the synergism observed could be attributed to non-specific damage to the bacterial membrane. This would be a strong indicator the compound would have similar activity against mammalian cells and hence would be cytotoxic. This was clearly the case for the EPI Phe-Arg-β-naphthylamide (PAβN; Marquez, 2005; Lomovskaya and Zgurskaya, 2011).

Therefore, there need to be a thorough investigation in order to verify true EPI action (**Figure 3**). Compounds that permeabilise the membrane of Gram-negative organisms will always show synergism with antibiotics. For example, the modulatory effect of α-tocopherol in multidrug resistant Gram-negative bacteria such as *P. aeruginosa* and *E. coli* could most probably be attributed to the effects of α-tocopherol on the membrane (Andrade et al., 2014). It is therefore important that potential inhibitors are not only identified on their synergism with antibiotics, but that a subsequent biochemical assays are performed to determine that the compounds are truly acting by inhibiting drug eﬄux.

In order to qualify as an EPI a compound must be able to satisfy the following criteria as stipulated by Lomovskaya et al. (2001).

(a) It must potentiate the activity of antibiotics to which a strain has developed resistance as a result of the expression of a drug eﬄux pump.
(b) It should not have an effect on sensitive strains which lack the drug eﬄux pump.
(b) It must not reduce the MIC of antibiotics which are not eﬄuxed.
(d) It must increase the level of accumulation and decrease the level of extrusion of compounds which are substrates of the eﬄux pump.
(e) It must not permeabilise the outer membrane.
(f) It must not affect the proton gradient across the inner membrane.

All the above criteria can be addressed with well-developed techniques as outlined below and in **Figure 3**, which would be amenable to scale-down for high throughput analysis.

### Measuring Synergism

The first thing to do is to determine the MIC of the test compound using standard broth dilution assays (Lomovskaya et al., 2001; Welch et al., 2010; Ohene-Agyei et al., 2012, 2014). Ideally the compound should not be toxic to bacterial cells or only toxic at high concentrations. This would prevent resistance against the test compound from developing very quickly. The compound would then be used at concentrations below its MIC (usually 4× lower than the MIC) to test for synergism with antibiotics to which the organism has developed resistance. Synergism is best studied using checkerboard assays. These assays could be performed in a 96-well plate format with the antibiotic serially diluted along the ordinate and the test compound serially diluted along the abscissa (Lomovskaya et al., 2001; Orhan et al., 2005; Ohene-Agyei et al., 2014). The MIC of the antibiotic is determined in the presence of a range of different concentrations of the compound. Antibiotic-EPI interactions are subsequently classified on the basis of fractional inhibitory concentration (FIC). The FIC index is the sum of the FIC of each of the antibiotics, which in turn is defined as the MIC of the antibiotic when used in combination divided by the MIC of the antibiotic when used alone. The combination is considered synergistic when the ΣFIC is ≤0.5, indifferent when the ΣFIC is >0.5 to <2, and antagonistic when the ΣFIC is ≥2.

### Ensuring the Compound has no Effect on Strains Which Lack the Drug Eﬄux Pump

An effective way of testing the effect of a compound on eﬄux pump mediated resistance is to use a wild-type antimicrobial resistant strain and a sensitive strain with a genomic deletion of the IMP. Checkerboard assays can be performed on the wild type strain to determine if MIC drop toward that of sensitive strain. Conversely the compound should not have an effect on the MIC of the sensitive strain.

However, it is important not to use a strain with a TolC deletion. TolC is a multi-functional protein that operates with the majority of MFP-dependent transporters encoded in the genome of *E. coli* (Zgurskaya et al., 2011). Results from TolC minus cells would therefore be complicated by effects which are not related to active drug eﬄux (Ohene-Agyei et al., 2014).

### Inhibition of Substrate Transport

The ability of a potential EPI to inhibit substrate transport in a drug eﬄux pump can be measured by performing substrate accumulation assays or by measuring substrate eﬄux in the absence/presence of the putative EPI. Many fluorescent compounds are also substrates for drug eﬄux pumps. If these compounds undergo a change in fluorescence when bound to DNA/membrane lipids they can be used to measure the eﬄux activity of drug transporters. Many fluorescent compounds fulfill this role and are frequently used to measure drug eﬄux; examples are Hoechst 33342, berberine, ethidium bromide, TMA-DPH [1-(4-trimethylammoniumphenyl)-6-phenyl-1,3,5-hexatriene p-toluenesulfonate], *N*-phenylnaphthylamine and Nile Red which display enhanced fluorescence intensity when accumulated inside the cell or doxorubicin and rhodamine 6G for which accumulation inside cells results in quenching of the fluorescence signal (Lee et al., 2001; Lomovskaya et al., 2001; Seeger et al., 2008a,b; Ohene-Agyei et al., 2012, 2014; Cha et al., 2014). In drug accumulation assays the difference in rate of accumulation of the fluorescent compound between cells with and without an active eﬄux pump are used as an indication of eﬄux, since eﬄux will result in lower accumulation of compound. In drug eﬄux assays, the de-energized cells are pre-loaded with the fluorescent compound and then energized by the addition of glucose to catalyze drug eﬄux (observed as a drop in fluorescence). Drug influx assays are more straightforward and much quicker to perform than drug-eﬄux assays as de-energization and pre-loading can be time consuming. In addition, all the samples must be pre-loaded to the same level of fluorescence to avoid differences in eﬄux rate as a result of differences in the concentration of drug inside the cell. The main drawback of using fluorescent compounds to measure the effect of an EPI on drug eﬄux is that the potential EPI could be highly colored or fluorescent itself and thus interfere with the measurement. Recently, Bohnert et al. (2010) developed a method using Nile Red for eﬄux which are compatible with highly colored or fluorescent compounds.

### Testing of Outer Membrane Permeabilization

The most effective method to measure outer membrane permeabilization is the nitrocefin hydrolysis method. Nitrocefin is a chromogenic β-lactam which changes from yellow (∼380 nm) to red (∼490 nm) when it is hydrolyzed by the periplasmic β-lactamase, hence nitrocefin hydrolysis can be followed by measuring the absorbance at 490 nm. If the test compound permeabilises the outer membrane, nitrocefin will diffuse more quickly over the membrane and hence the rate of nitrocefin hydrolysis will increase as a result (Lomovskaya et al., 2001; Ohene-Agyei et al., 2014). It is important to perform these essays in the presence of the ionophore CCCP to de-energize cells and prevent nictrocefin eﬄux.

### Testing of Inner Membrane Permeabilization

Several methods exist to measure permeabilization of the inner-membrane. A DNA stain which does not penetrate the membrane of intact bacterial cells and which will undergo an increase in fluorescence quantum yield when bound to DNA such as propdium iodide or SYTOX Green could be used (Roth et al., 1997; Nakashima et al., 2011). SYTOX Green would be preferred for its sensitivity as it undergoes a >500-fold enhancement in fluorescence emission when bound to DNA.

Other methods to measure the intactness of the bacterial inner membrane involve the use or measurement of the pmf in *E. coli*. Opperman et al. (2014), employed an assay based on the uptake of [*methyl*-^3^H]β-D-thiogalactopyranoside ([^3^H]TMG) by the LacY permease. The activity of the lactose permease is dependent on the pmf as it catalysis substrate/H^+^ symport. Lomovskaya et al. (2001) probed the intracellular pH of *E. coli* cells by measuring the nuclear magnetic resonance (NMR) of the ^31^P in the inner-membrane. Although both these two methods are effective they are quite time consuming and require access to specialist equipment. The magnitude of the individual components of the pmf can be measured directly by a simple fluorescence assay utilizing the fluorescent membrane potential probe 3,3′-diethyloxacarbocyanine iodide (DIOC_2_(3); Venter et al., 2003). Moreover, the DIOC_2_(3) assay can easily be adapted to 96-well format for the quick analysis of test compounds on the inner membrane in high-throughput screening.

### Use of a Non-Substrate

Another way of ruling out false positives and establishing that compounds do not act non-specifically is to measure the effect of the test compound on an antibiotic which is not an eﬄux pump substrate. For example our group used rifampicin, which is not transported by the AcrAB-TolC drug eﬄux pump from *E. coli* (Ohene-Agyei et al., 2014). The test compounds should not lower the MIC of rifampicin. Any reduction in the MIC of rifampicin would indicate that the compound does not potentiate antibiotic action by inhibition of eﬄux, but acts by indirect means such as permeabilization of the membrane.

## EPIs Against Gram-Negative Bacteria Identified so Far

The first EPI to be identified against RND pumps in Gram-negative bacteria was the peptidomimetic PAβN, originally referred to as MC-2077110. PAβN was identified in a screen for levofloxacin potentiators against resistant *P. aeruginosa*. Unfortunately, in addition to eﬄux pump inhibition it also permeabilized the outer membrane (Lomovskaya et al., 2001). Derivatives of PAβN with reduced toxicity, enhanced stability, and better solubility were developed and advanced to the pre-clinical stage, however, failed due to toxicity issues (Marquez, 2005; Lomovskaya et al., 2006; Lomovskaya and Zgurskaya, 2011; Bhardwaj and Mohanty, 2012).

The structural basis for the inhibition of the RND transporters has been recently described with the publication of the crystal structures of AcrB from *E. coli* and MexB from *P. aeruginosa* bound to a pyridopyrimidine derivative D13–D900 (Nakashima et al., 2013). The inhibitor binding almost overlapped with the binding of the substrates minocycline and doxorubicin, while part of the inhibitor inserted into a narrow phenylalanine rich region in the deep binding pocket, termed the hydrophobic trap by the authors. The authors suggested that the inhibitor competitively inhibit substrate binding and hinders the functional rotation of the eﬄux pumps.

As there is only one structure of a RND protein bound to an inhibitor published to date, docking, and molecular simulation studies were used to investigate the putative binding modes of other inhibitors such as PAβN and NMP (Vargiu et al., 2014) while *in silico* screening also provided information on the binding of putative EPIs (Ohene-Agyei et al., 2014). Both PAβN an NMP were predicted to interact with the switch loop while D13–D9001and MBX2319 have more interactions with the hydrophobic trap first identified by Nakashima et al. (2013).

**Table 1** summarizes the compounds reported to act as EPIs against Gram-negative organisms so far. The term EPI is used loosely here as some of the included compounds were identified based on their synergism with one or more antibiotic while no further analysis was performed to study the mechanism of inhibition or rule out non-specific effects such as membrane permeabilization.

**Table 1:** Eﬄux pump inhibitors (EPIs) against Gram-negative pathogens.

Compound	Source	Protein/ Organism	Actions^1^	Essays performed	Reference
**Synthetic Compounds**
Phe-Arg-β-naphthylamide (PAβN; MC-207,110)	Synthetic	MexAB-OprM, MexCD-OprJ, MexEF-OprN *(Pseudomonas aeruginosa)*	Synergise with fluoroquinolones	Antibacterial Synergism Substrate accumulation Inhibition of eﬄux Effect on outer-membrane	Lomovskaya et al. (2001)
7-nitro-8-methyl-4-[2′-(piperidino)ethyl] aminoquinoline	Alkylamino-quinolines	AcrAB-TolC *(Enterobacter aerogenes*)	Reduced MIC of Cam, Nor, and Tet Increased Cam uptake	Antibacterial Synergism Substrate accumulation	Mallea et al. (2003)
2,8-dimethyl-4-(2′-pyrrolidinoethyl)-oxyquinoline	Alkoxy-quinoline derivative	*E. aerogenes Klebsiella pneumonia*	Reduced MIC of Nor, Tet, Cam	Substrate accumulation Effect on membrane	Chevalier et al. (2004)
1-(1-Naphthylmethyl)-piperazine (NMP)	Synthetic	AcrAB, AcrEF (*Escherichia coli*)	Reduction in MICs of Lev, Oxa, Rif, Cam, Clr Increased accumulation of ethidium	Antibacterial Substrate accumulation	Kern et al. (2006)
New chloroquinoline derivatives	Fluoroquinolones	AcrAB-TolC (*E. aerogenes)*	Reduced MIC of Cam	Antibacterial Substrate accumulation	Ghisalberti et al. (2006)
3-amino-6-carboxyl-indole, 3-nitro-6-amino-indole	Designed and synthesized based on TolC structure	AcrAB-TolC*(E. coli)*	Reduced MIC of cam, tet, ery, and cip	Antibacterial Synergism	Zeng et al. (2010)
4-(3-morpholinopropylamino)-quinazoline	4-alkylaminoquinazoline derivatives	AcrAB-TolC MexAB-OprM *(E. coli P. aeruginosa)*	Reduced MIC of Cam, Nal, Nor, and Spfx Increased Cam uptake	Antibacterial Synergism Substrate accumulation	Mahamoud et al. (2011)
MBX2319	S*ynthetic* pyranopyridine	AcrB *(E. coli)*	Decreased MIC of Cip, Lev, and Prl	Docking Time kill assay Substrate accumulation Effect on outer-membrane Effect on inner-membrane	Vargiu et al. (2014), Opperman et al. (2014)
2-substituted benzothiazoles	S*ynthetic*	AdeABC *(Acinetobacter baumannii*)	Reduced MIC of cip	Pharmacophore hypothesis	Yilmaz et al. (2014)
**Natural Compounds**
EA-371α and EA-371δ	*Streptomyces* MF-EA-371-NS1	MexAB-OprM *(P. aeruginosa)*	Reduce MIC of Lev	Synergism Substrate accumulation	Lee et al. (2001)
Geraniol	*Helichrysum italicum*	*E. coli P. aeruginosa A. baumanii*	Reduced MIC of β-lactams, quinolones, and Cam	Antibacterial Synergism	Lorenzi et al. (2009)
Plumbagin	*Plumbago indica*	AcrB (*E. coli*)	Reduced MIC of Ery, Cam, TPP, SDS, tet Inhibition of Nile Red eﬄux	*In silico* screening Antibacterial Synergism Non-substrate control Inhibition of eﬄux Effect on outer-membrane	Ohene-Agyei et al. (2014)
Nordihydroguaretic acid (NDGA)	Creosote bush	AcrB (*E. coli*)	Reduced MIC of Ery, Cam, Nov, Tet, and TPP		
Shikonin	*Lithospermum erythrorhizon*	AcrB (*E. coli*)	Reduced MIC of TPP		
(-)-epigallocatechin gallate EGCG	Green tea	*Campylobacter* spp.	Reduced MIC to Ery and Cip	Antibacterial Synergism	Kurincic et al. (2012)
Curcumin	*Curcuma* longa (Zingiberaceae)	*P. aeruginosa*	Reduced MIC Mem, Carb, Caz, Gen, and Cip	Antibacterial Synergism	Negi et al. (2014)
Lanatoside C and diadzein	*Phytochemical*	*AcrB, MexB (E. coli, P. aeruginosa)*	Reduced MIC of Lev and Carb Increased accumulation of EtBr	High-throughput virtual screening Synergism Substrate accumulation	Aparna et al. (2014)
4-hydroxy-α-tetralone	*Ammannia sp*	*E. coli*	Reduced MIC of Tet	RT-PCR study *In silico* docking	Dwivedi et al. (2014)
**Non-antibacterial drugs**
Trimethoprim and Epinephrine	Small heterocyclic or nitrogen-containing drugs	*S. typhimurium* *E. cloacae* *S. marcescens* *P. aeruginosa* *K. pneumoniae* *E. coli*	Reduced MIC of Cip	Antibacterial Synergism Substrate accumulation Growth kinetics	Piddock et al. (2010)
Chlorpromazine, Amitryptiline, Trans-chlorprothixene	Non-antibiotic drugs	*P. aeruginosa*	Reduced MIC of Pen, Cxm, and Tob	Antibacterial Synergism	Kristiansen et al. (2010)
Sertraline	Selective Serotonin Re-uptake Inhibitors	AcrAB, AcrEF, MdtEF, and MexAB	Inhibition of Nile Red eﬄux	Inhibition of eﬄux RT-PCR	Bohnert et al. (2011)
Artesunate	Anti-malarial drug	AcrAB-TolC *(E. coli)*	Reduced MIC ofβ-lactam antibiotic Increased Dau uptake Reduce mRNA expression	Antibacterial Synergism Substrate accumulation RT-PCR	Li et al. (2011)
Pimozide	Neuroleptic drug	AcrAB-TolC (*E. coli)*	Reduced MICs of Oxa and EtBr Inhibition of Nile rRed eﬄux	Synergism Substrate eﬄux	Bohnert et al. (2013)

*^1^Abbreviations used: Cam, Chloramphenicol; Carb , Carbanecillin; Caz , Ceftazidime; Cip , Ciprofloxacin; Clr , Clarithromycin; Cxm , Cefuroxime; Dau , Daunomycin; Ery , Erythromycin; EtBr , Ethidium Bromide; Gen, Gentamicin; Lev , Levofloxacin; Mem , Meropenem; Nal , Nalidixic acid; Nor , Norfloxacin; Oxa , Oxacillin; Pen , Penicillin; Prl , Piperacillin; Rif , Rifampicin; Spfx , Sparfloxacin; Tet , Tetracycline; Tob , Tobramycin; TPP , Triphenylphosphonium*.

## Conclusion

There are various papers reporting the ability of crude extracts from plants or other organisms to reduce antibiotic resistance that were not dealt with in this review. As can be seen from **Table 1**, there is also a sizable amount of pure compounds which were able to synergise with antibiotics against drug resistant Gram-negative bacteria. However, the translation of these promising compounds into EPIs for clinical application is still lacking. The most probable reason for the discrepancies in lead compounds and final outcome is the deficiency of follow through from first identification of a compound with synergistic effects to identification of true EPI activity and providing a thorough investigation into mechanism of action. With this review we aimed to summarize the current knowledge of how drug eﬄux can be inhibited.

The tools necessary to identify, test and characterize the mechanism of action of a putative EPI were also provided in order to aid the discovery and development of EPIs with which we would be able to stem the tide of multidrug resistant Gram-negative infections.

## Conflict of Interest Statement

The authors declare that the research was conducted in the absence of any commercial or financial relationships that could be construed as a potential conflict of interest.
